# A caspase-6-cleaved fragment of Glial Fibrillary Acidic Protein as a potential serological biomarker of CNS injury after cardiac arrest

**DOI:** 10.1371/journal.pone.0224633

**Published:** 2019-11-06

**Authors:** Ditte S. Jonesco, Christian Hassager, Martin Frydland, Jesper Kjærgaard, Morten Karsdal, Kim Henriksen

**Affiliations:** 1 Biomarkers & Research, Nordic Bioscience, Herlev, Denmark; 2 Department of Cardiology B, Copenhagen University Hospital, Rigshospitalet, Copenhagen, Denmark; Nathan S Kline Institute, UNITED STATES

## Abstract

Blood levels of Glial Fibrillary Acidic protein (GFAP) reflect processes associated with different types of CNS injury. Evidence suggests that GFAP is cleaved by caspases during CNS injury, hence positioning GFAP fragments as potential biomarkers of injury-associated processes. We set out to develop an assay detecting the neo-epitope generated by caspase-6 cleavage of GFAP (GFAP-C6), and to assess the ability of GFAP-C6 to reflect pathological processes in patients suffering a cardiac arrest and subsequent global cerebral ischemia. Anti-GFAP-C6 antibodies recognized their specific target sequence, and dilution and spike recoveries in serum were within limits of ±20% reflecting high precision and accuracy of measurements. Intra- and inter-assay CVs were below limits of 10% and 15%, respectively. Serological levels of GFAP-C6 were significantly elevated 72 hours after CA (Mean±SD) (20.39±10.59 ng/mL) compared to time of admission (17.79±10.77 ng/mL, p<0.0001), 24 hours (17.40±7.99 ng/mL, p<0.0001) and 48 hours (17.87±8.56 ng/mL, p<0.0001) after CA, but were not related to neurological outcome at day 180. GFAP-C6 levels at admission, 24, 48, and 72 hours after cardiac arrest correlated with two proteolytic fragments of tau, tau-A (r = 0.30, r = 0.40, r = 0.50, r = 0.53, p < 0.0001) and tau-C (r = 54, r = 0.48, r = 0.55, r = 0.54, p < 0.0001), respectively. GFAP-C6 levels did not correlate with other markers of CNS damage; total tau, NSE and S100B. In conclusion, we developed the first assay detecting a caspase-6 cleaved fragment of GFAP in blood. Increased levels at 72 hours after cardiac arrest as well as moderate correlations between GFAP-C6 and two other blood biomarkers of neurodegeneration suggest the ability of GFAP-C6 to reflect pathological processes of the injured brain. Investigations into the potential of GFAP-C6 in other types of CNS injury are warranted.

## Introduction

Astrocytes are a predominant type of specialized glial cell in the CNS, providing metabolic and trophic support of neurons and assisting in synaptic transmission[1]. Activation of astrocytes is a prominent feature of traumatic brain injury (TBI), cerebral ischemia, as well as neurodegenerative diseases[1–3]. Concurrent upregulation of Glial Fibrillary Acidic Protein (GFAP), which is the main constituent of intermediate filaments in astrocytes occurs[1]. As a consequence, intensive focus has been put on GFAP and its unspecified breakdown products (GFAP-BDPs) as possible markers of different types of injury to the CNS [4,5,14,15,6–13]. Several studies have found GFAP levels to be elevated in CSF and blood of patients with mild to severe Traumatic Brain Injury (TBI) and levels of GFAP reflect severity of injury [4–9]. Similarly, publications on serological levels of GFAP after CA report increased levels after injury [10,11] with the ability to separate good from poor neurological outcome 12 hours after CA [12]. Also, CSF levels of GFAP are known to differentiate between patient with ischemic stroke and healthy individuals within the first 24 hours after injury, and GFAP correlates to severity of stroke [14,15]. Clearly, alterations in GFAP levels reflect processes associated with different types of injury to the CNS. The degree of detail on processes underlying CNS injury, provided by a biomarker, might increase by targeting disease-specific posttranslational modifications (PTM) of proteins as biomarkers. Applying PTMs as markers of disease has proved beneficial before. An example is seen in Alzheimer’s Disease (AD) where not only total tau but also phosphorylated tau and the γ-secretase-cleaved APP fragment, Aβ_42_, is applied in the diagnostic and prognostic workup [16]. Likewise, in Alexander disease, a rare neurodegenerative disease characterized by cytoplasmic proteinaceous aggregates containing GFAP, the proteolytic fragmentation of GFAP has been related to disease severity [17] and has been suggested to be central in the pathogenesis [17,18]. Fragmentation of proteins by caspases is one possible process resulting in PTMs [18–21]. Caspase-6 cleavage of GFAP is observed in vitro, resulting in a C-terminal fragment unable to assemble into filaments as well as an N-terminal fragment prone to aggregation [18]. Cleavage of GFAP by caspases is also observed in both apoptotic and non-apoptotic astrocytes ex vivo [19–21]. In non-apoptotic astrocytes, caspases are believed to aid in the rearrangement of the cytoskeleton as these cells become reactive [19,22]. Involvement of caspases in cytoskeleton rearrangement is also evident within neurons [23–26]. In vitro and in vivo investigations suggest that astrocytes undergo apoptosis during neurodegenerative disease, TBI and cerebral ischemia [20,27–31]. Furthermore, it is suggested that caspases are involved in this process [20,28,29,32,33]. Pro-caspase-6 is observed in astrocytes after transient focal cerebral ischemia in rats [32] and active caspase-6 localizes with GFAP in the brain of patients with HIV-associated dementia [32,33]. We hypothesize that caspase-6-generated fragments of GFAP (GFAP-C6) could reflect pathological processes underlying neurodegeneration as a function of ischemia of the brain resulting from a cardiac arrest (CA). To test our hypothesis, we set out to develop a highly specific and technically robust serum-based enzyme-linked immunosorbent assays (ELISAs) for detection of the neo-epitope generated by caspase-6 cleavage of GFAP, GFAP-C6. We then determined the levels of a GFAP-C6 in serum of CA patients with global cerebral ischemia. Serum samples were obtained from a single-center, prospective, observational sub-study comprising 171 patients enrolled at Copenhagen University Hospital, Rigshospitalet in the TTM-Trial (November 2010 to July 2013) [34]. GFAP-C6 levels in serum, collected at four time points following CA were assessed in relation to neurological outcome after 180 days as well as other blood biomarkers, patient CA data and demographics.

## Material and methods

### Reagents and peptides

All chemicals were purchased from Merck (Whitehouse Station, NJ, USA) and Sigma-Aldrich (St. Louis, MO, USA). Monoclonal antibodies (mAb) were produced and validated using synthetic peptides: a) Immunogenic peptide NLAQDLATVR-GGC-KLH (Keyhole-Limpet-Hemocyanin), b) screening peptide NLAQDLATVR-biotin, c) selection peptide NLAQDLATVR and d) elongated peptide **D**NLAQDLATVR, from Scilight Biotechnology LLC (Beijing, China) as well as e) non-sense selection peptide AGRKRQSLQF and f) non-sense screening peptide biotin-AGRKRQSLQF from Genscript (Nanjing, China).

### Production of mAbs with high specificity for GFAP-C6

Anti-GFAP-C6 mAb was raised against the amino acid sequence ^143^NLAQDLATVR^152^ corresponding to the N-terminal of the GFAP fragment generated by caspase-6 cleavage at Asn^143^. The neo-epitope was blasted for homology to other proteins using the “NPS@: Network Protein Sequence Analysis with the UniprotKB/Swiss-prot database” software online [35]. Anti-GFAP-C6 mAb was produced by s.c. immunization of 4-6-week-old Balb/C female mice with 200 μL emulsified antigen containing 50 μg immunogenic peptide. After consecutive immunizations, the mouse displaying the highest antibody titer was boosted by injecting 50 μg immunogenic peptide in 100 μl 0.9% NaCl solution intravenously. The spleen was isolated for cell fusion and spleen cells were fused with SP2/0-Ag14 mouse myelomas, to produce immortal antibody-producing hybridoma. The fusion cells were cultured in 96-well plates at 37°C with 5% CO_2_. Monoclonal growth was ensured by standard limited dilution. Clones producing mAb specifically recognizing the epitope sequence (NLAQDLATVR) but not an elongated sequence (**D**NLAQDLATVR) were selected for sub-cloning. Last, monoclonal hybridoma were expanded and antibodies purified from hybridoma supernatant using a prepacked HiTrap^TM^ Protein G Sepharose column (GE Healthcare Life Science, Buckinghamshire, UK). Mice were housed under standardized conditions (20–23°C, 30–60% relative humidity, and a 12-h/12-h light/dark cycle) and were caged five mice per cage in cage type lllH (1291H, 425x266x185mm, floor area 800cm2). They had access to food and water ad libitum, and the general conditions were monitored daily by trained animal technicians. Red bumps after injection were expected. However, if a skin infection or tumor was found at the sites of injection, the mouse would be terminated to reduce suffering and pain. Mice were sacrificed by cervical dislocation. All the work on mice was approved by Beijing laboratory animal administration office and animal ethics committee of Nordic Bioscience (Beijing).

### Development of ELISAs specifically detecting GFAP-C6

A competitive ELISA was developed based on our mAbs, allowing specific detection of GFAP-C6. Optimal buffers, incubation time and temperature as well as antibody and antigen concentrations, were identified. The final settings for the ELISA were as follows: A 96-well ELISA plate pre-coated with streptavidin (cat. no.11940279, Roche, Basel Switzerland) was further coated with 2.5 ng/mL screening peptide dissolved in assay buffer (25 mM TBS-BTB, 2 g/L NaCl, pH 7.4) for 30 min at 20°C, 300 rpm. The plate was washed five times in wash buffer (20 mM Tris, 0.1% Tween-20, 50 mM NaCl, pH 7.2). 20 μL/well of standard peptide or sample was added followed by 100 μL/well of the anti-GFAP-C6 mAb diluted in assay buffer. The plate was incubated for one h at 20°C, 300 rpm before washing five times in wash buffer. 100 μL/well of horseradish peroxidase (HRP)-conjugated anti-mouse antibodies diluted in assay buffer was added and the plate incubated for one hour at 20°C, 300rpm. Again, the plate was washed five times. Then, 100 μL/well tetramethylbenzidine (TMB) (cat. no. 4380, Kem-En-Tec, Taastrup, Denmark) was added and the plate incubated for 15 min at 20°C, 300 rpm. The colorimetric reaction was stopped by adding 100 μL/well of stopping solution (1% H_2_SO_4_). The plate was measured at 450 nm with 650 nm as the reference (SpectraMax M; Molecular Devices, CA, USA). A standard curve was produced by serial dilution of the selection peptide and plotted using a four-parameter curve fit model. Standard concentrations were: 0.09, 0.37, 1.47, 5.86, 23.44, 93.75, 375.00, 1500.00 ng/mL. Data were analysed using the SoftMax Pro v.6.3 software.

#### Technical validation

Specificity of the anti-GFAP-C6 mAbs in the context of the GFAP-C6 ELISA was investigated. This was done by performing the established ELISA procedure using a non-sense screening-peptide as well as serial dilutions of non-sense selection- and elongated- peptide sequences. Linearity of dilution was determined by dilution of five human serum samples and calculated as the recovery percentage of undiluted sample. Accuracy of the assay was assessed by the recovery percentage of spiking. Six human serum samples of known GFAP-C6 concentration were spiked with six other human serum samples with known GFAP-C6 concentrations. The recovery percentage was calculated as the actual concentration over the expected concentration of spiked samples. Inter- and intra-assay precision was assessed by performing 10 independent analytical runs measuring duplicates of five different serum samples and calculating the mean CV%. In order to assess possible interference in the ELISA from endogenous analytes, high/low content of lipemia/lipids, (4.83/10.98 mM) and biotin (3.00/9.00 ng/mL) as well as high content of hemoglobin (0.31mM) were added to two different serum samples of known concentrations. Recovery percentage was calculated with the normal serum samples as reference. Analyte stability was assessed by exposing serum samples to repeated freeze/thaw cycles as well as different temperatures for various time intervals. First, GFAP-C6 concentrations were determined in three different samples in four freeze/thaw cycles and the recovery was calculated with the zero cycle as a reference. Second, three samples were stored at either 4°C or 20°C for 0, 2, 4, 24, and 48 h before determining GFAP-C6 concentrations and recovery was calculated with 0 h as a reference. The Lower Limit of Detection (LLOD), Lower Limit of Quantification (LLOQ) and Upper Limit of Detection (ULOD) define the assay range within which quantification of serum is made with high accuracy and precision. The LLOD was determined from 76 measurements of zero samples (assay buffer) and calculated as the mean OD– 3 standard deviations. The OD value was then interpolated into ng/mL based on the standard curve parameters. The lowest possible analyte concentration that can be quantified with acceptable accuracy and precision is represented by the LLOQ. To determine this value, three independent assay runs measuring quadruplicates of two serial diluted serum samples were performed. A total impression score was calculated for each dilution step taking three parameters into account: the analyte recovery as well as inter- and intra-variations. The lowest possible concertation of analyte with an impression score below 25% equals the LLOQ. The ULOD was established based on 10 independent determinations of the highest standard peptide concentration and was calculated as mean–three standard deviations.

#### Cleavage of human GFAP

*In vitro* cleavage of human GFAP with recombinant human caspase-6 was performed. First GFAP was dissolved in dH_2_O at a concentration of 1 mg/mL and caspase-6 was dissolved in PBS with 15% glycerol at a final concentration of 3.33 units/μL. Protein and enzyme was mixed in cleavage buffer (50 mM HEPES, 50mM NaCl, 10 mM EDTA, 10mM DTT, 0.1% (w/v) Chaps, 5% (v/v) Glycerol, pH: 7.2) in a ratio of 1.2:1 and incubated at 37°C for 24h. Cleavage was ended by placing the solutions at -80°C. Solutions were tested in the GFAP-C6 ELISA.

#### Biological and clinical validation of GFAP-C6

GFAP-C6 was measured in serum samples from a prospective, observational sub-study comprising 171 patients enrolled at the Copenhagen University Hospital, Rigshospitalet in the TTM-Trial (November 2010 to July 2013). The Ethics Committee of the Capital Region of Copenhagen approved the main study protocol (H-1-2010-059). The primary goal of the TTM-trial was to investigate the benefit of target temperature management. Details on trial design, statistical analysis plan and results have been published [34,36,37]. Briefly, included in the study were comatose patients, 18 years of age or older, admitted after out-of-hospital cardiac arrest of presumed cardiac cause. Exclusion criteria were: Unwitnessed asystole as the primary rhythm, time between return of spontaneous circulation (ROSC) and screening > 4 hours, intracranial bleeding suspected or confirmed, temperature on admission < 30°C. Patients were randomly assigned to two temperature regimes of 33°C or 36°C for 24 hours after CA. Patients were sedated, intubated and mechanically ventilated. After 36 hours, sedation was lifted and the patients allowed to recover spontaneously. Neurological outcome after 180 days was defined as good or poor based on scores of the cerebral performance category (CPC) scale (CPC 1–2 = good; CPC = 3–5 = bad). CPC 1, represents good cerebral performance or minor disability; 2, moderate disability; and 3, severe disability; 4, coma or vegetative state; and 5, death. Assessment of the CPC score was performed blinded according to the TTM trial protocol [34]. Blood serum was collected 0, 24, 48 and 72 hours after ROSC and samples were aliquoted and placed at -80°C for later analysis. The primary end-point of this sub-study was neurological outcome at day 180. We did not distinguish between patients assigned to the two temperature regimes, as the target temperature management at 33°C was comparable to that of 36°C [36].

### Statistical analysis

Differences between groups were tested using the Student t-test or the Mann-Whitney U test. Associations between variables were assessed using Spearman’s rho correlation coefficients. The kinetics of GFAP-C6 were assessed using One-way ANOVA with repeated measures. Adjustment for multiple comparisons was performed with Dunn's multiple comparisons test. Serum GFAP-C6 levels in patients with good versus bad outcome at different time points after CA were compared using the Two-way ANOVA with repeated measures. Assumptions were checked and met. Results are shown as Line plots with error bars or Scatter dot plots with the mean or median and 95% CI indicated. The significant level was set at 0.05. Asterisks indicate the following: *P < .05; **P < 0.01; ***P < 0.001; ****P < 0.0001. Graphs and statistical analyses were performed using GraphPad Prism version 7 (GraphPad Software, Inc., CA, USA)

## Results

### The GFAP-C6 ELISA is highly specific for the target sequence

**Fig 1:**
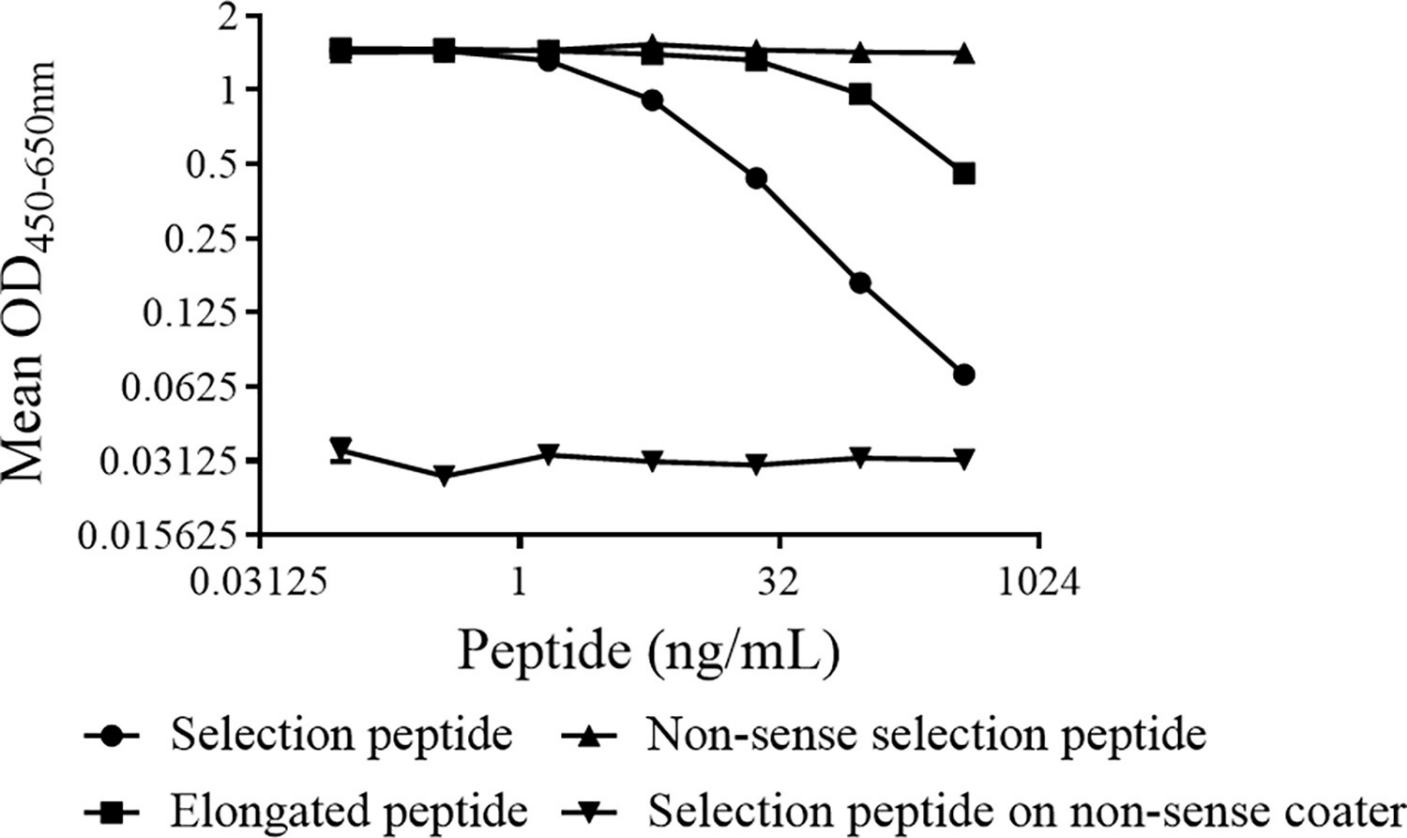
Peptide specificity test of monoclonal anti-GFAP-C6 antibodies. Shown are the binding of antibodies to selection-, elongated-, non-sense selection- and non-sense coater peptides of concentrations within the measuring range of each assay. An addition of a single amino acid (elongated peptide) disrupted the binding to a satisfying extent. Non-sense peptides were completely imperceptible to the antibodies. Shown are the mean of two determinations plotted with the standard deviation.

**Fig 2:**
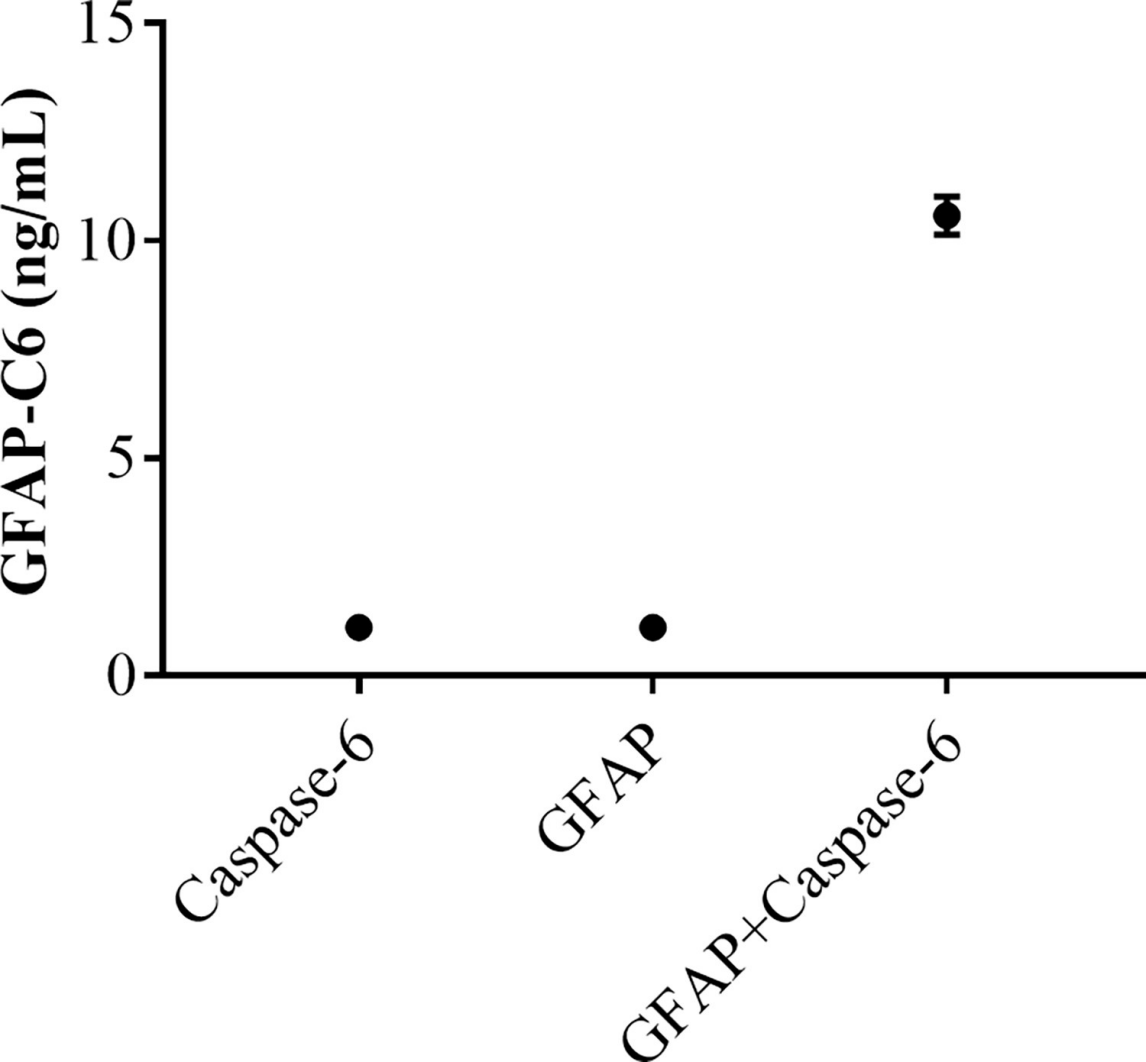
Specificity test of GFAP-C6 ELISA. Shown are the signal obtained by GFAP-C6 ELISA in response to caspase-6 cleaved rhGFAP, non-cleaved rhGFAP, caspase-6 and cleavage buffer incubated at 37°C for 24 h. Concentrations below LLOD were given the value of LLOD. Shown are the mean of two determinations plotted with the standard deviation.

To assess the specificity of the GFAP-C6 ELISA, we performed several investigations. First, we blasted the neo-epitope sequence for homology to other proteins and found no homology with other protein sequences than that of GFAP (S1 Fig). Subsequently we compared a dilution series of selection peptide with that of elongated peptide, nonsense selection peptide and nonsense screening peptide dilution series. Adding a single amino acid at the N-terminal of the neo-epitope sequence disrupts the binding of anti-GFAP-C6 antibodies to a large extent. Likewise, dilution series of the non-sense selection peptide were not recognized by the antibodies, while coating the plate with non-sense screening peptides resulted in complete lack of signal (Fig 1). Lastly, in order to challenge the GFAP-C6 mAbs with fragments displaying a natural tertiary structure resembling the endogenous GFAP-C6 to a larger extent than synthetic peptides, we produced caspase-6-cleaved rhGFAP fragments *in vitro* and applied these in the GFAP-C6 ELISA. The assay only detected caspase-6 cleaved rhGFAP and not full-length rhGFAP, caspase-6 or cleavage buffer (Fig 2). The results of these investigations demonstrate high target specificity of the antibodies as applied in the GFAP-C6 ELISA.

### The GFAP-C6 ELISA is technically robust with high accuracy and precision

The assay range, defined as the LLOQ and the ULOD, was determined to be 2.16ng/mL– 247.38ng/mL. The LLOD was determined to be 1.12ng/mL and the mean IC50 value was 8.41ng/mL. Diluting samples 1+1 to 1+5 allowed linearity of analyte dilution with recovery percentages within 100±20% (Table 1). The ELISA showed high accuracy with a mean spiking recovery of 106% using high analyte serum samples (Table 2). Recovery percentages of GFAP-C6 levels in serum samples spiked with low and high levels of the common endogenous serum analytes, biotin, lipid, and hemoglobin, show no interference from these analytes (Table 3). Mean recovery percentages for low (3.00ng/mL) and high (9.00ng/mL) biotin are 102%. For low (4.83mM) and high (10.98mM) levels of lipid the mean recovery percentages are 93% and 90%, respectively, and for high (0.31mM) levels of hemoglobin mean recovery percentage is 93% (Table 3). Furthermore, stability of GFAP-C6 analyte was not affected by 1, 2, 3 or 4 freeze/thaw cycles as shown by recovery percentages of GFAP-C6 at 98%, 96%, 103%, and 102%, respectively (Table 4). Further analysis of analyte stability was performed by placing serum samples at 4°C or 20°C for 4, 24, and 48 hours before measuring GFAP-C6 concentrations. Mean recovery percentages of GFAP-C6 levels in samples placed at 4°C for 4, 24, and 48 hours were 97%, 99%, and 90%, respectively (Table 5). Mean recovery percentages of GFAP-C6 levels in samples placed at 20°C for 4, 24, and 48 hours were 88%, 80%, and 63%, respectively (Table 5). Thus, stability of GFAP-C6 analyte is affected only if stored at 20°C between 24 and 48 hours. Inter- and intra-assay variation were determined to be 10% and 4%, respectively, reflecting high technical stability of the GFAP-C6 ELISA (Table 6). An overview of the technical parameters is given in Table 7.

**Table 1:** Dilution recovery of GFAP-C6 ELISA.

Dilution recovery of human serum analyte						
Sample name	Dilution	GFAP-C6 (ng/mL)	%RE from 1+0	%RE from 1+1	%RE from 1+2	%RE from 1+3
1	1+0	9.12				
	1+1	6.24	137			
	1+2	3.82	126	92		
	1+3	2.82	124	91	99	
	1+4	2.45	134	98	107	108
	1+5					
2	1+0	8.88				
	1+1	7.40	167			
	1+2	3.74	126	76		
	1+3	2.68	121	73	96	
	1+4	2.34	132	79	104	109
	1+5	2.48	168	101	133	139
3	1+0	11.06				
	1+1	9.42	170			
	1+2	6.03	163	96		
	1+3	3.96	143	84	88	
	1+4	3.17	143	84	88	100
	1+5					
4	1+0	8.08				
	1+1	5.30	131			
	1+2	2.81	104	80		
	1+3					
	1+4	2.07	128	98	123	
	1+5	1.50	112	85	107	
5	1+0	4.22				
	1+1	4.27	203			
	1+2	2.23	159	78		
	1+3	1.60	152	75	96	
	1+4	1.35	160	79	101	105
	1+5	1.27	181	89	114	119
**Mean**			**144**	**86**	**104**	**111**

Going left to right, the columns contain information on: the extent of dilution, concentrations of GFAP-C6 in diluted serum samples, percent recovery of GFAP-C6 in serum diluted from 1+0 to 1+3. RE indicates recovery.

**Table 2:** Spiking recovery of in GFAP-C6 ELISA.

Spiking recovery of human serum analyte						
Sample name	Serum (ng/mL)	Spike (ng/mL)	Expected spiked serum (ng/mL)	Measured spiked serum (ng/mL)	%RE	%Mean RE
2	4.63	6.99	11.63	12.69	109	96
3	3.49	5.48	8.96	8.55	95	
4	4.89	6.80	11.69	10.73	92	
5	4.38	3.51	7.89	6.56	83	
6	2.94	5.54	8.48	7.63	90	
7	1.71	3.44	5.15	5.58	108	
2	4.63	2.83	7.46	9.78	131	110
3	3.49	2.80	6.29	6.25	99	
4	4.89	3.66	8.55	8.38	98	
5	4.38	1.48	5.86	5.84	100	
6	2.94	2.55	5.49	5.84	106	
7	1.71	1.53	3.25	4.16	128	
3	3.49	1.33	4.82	5.40	112	112
**Mean**						**106**

Going left to right, the columns contain information on: measured concentrations of GFAP-C6 in serum samples, measured concentration of GFAP-C6 in serum samples for spiking, expected concentration of spiked serum samples, measured concentration of spiked serum samples, percent recovery of GFAP-C6 analyte and mean percent recovery in all samples. RE indicate recovery.

**Table 3:** Interference of endogenous analytes in GFAP-C6 ELISA.

Interference of endogenous analytes								
	Hemoglobin		Biotin			Lipid		
	Ctrl	High	Ctrl	High	Low	Ctrl	High	Low
**Serum sample 1**								
GFAP-C6 ng/mL	8.12	7.66	8.20	8.89	8.41	8.75	8.54	7.95
%RE of Ctrl	100	94	100	108	103	100	98	91
**Serum sample 2**								
GFAP-C6 ng/mL	5.03	4.58	4.80	4.60	4.83	5.25	4.66	4.66
%RE of Ctrl	100	91	100	96	101	100	89	89
**Mean %RE**		**93**		**102**	**102**		**93**	**90**

Going left to right, the columns contain information on measured concentrations of GFAP-C6 in serum samples spiked with: Ctrl or high levels of hemoglobin, Ctrl, high or low levels of Biotin and Ctrl, high or low levels of Lipid. Going from top to bottom, rows contain information on serum sample ID, GFAP-C6 concentration in serum spiked with hemoglobin, biotin, lipid analytes and lastly % recovery from the Ctrl sample. Ctrl indicate control; RE, recovery.

**Table 4:** Stability of GFAP-C6 during repeated freeze/thaw cycles.

Analyte stability–Freeze/Thaw									
	GFAP-C6 (ng/mL)								
Sample name	0 cycle	1 cycle	%RE	2 cycle	%RE	3 cycle	%RE	4 cycle	%RE
1	6.52	6.14	94	6.83	105	7.08	109	7.06	108
2	3.84	4.04	105	3.59	93	4.05	105	4.02	104
3	9.58	9.12	95	8.58	90	9.03	94	8.96	94
**Mean %ER**			**98**		**96**		**103**		**102**

Going left to right, the columns contain information on measured concentrations of GFAP-C6 in serum samples exposed to freeze/thaw cycles: 0, 1, 2, 3 and 4 times. The %recovery from the 0 cycle sample after repeated freeze/thaw cycles is depicted adjecent to each freeze/thaw cycle. RE indicates recovery.

**Table 5:** Stability of GFAP-C6 as a function of time and temperature.

Analyte stability–temperature							
Sample name	GFAP-C6 (ng/mL)						
4°C	T: 0 hours	T: 4 hours	%RE from T: 0	T: 24 hours	%RE from T: 0	T: 48 hours	%RE from T: 0
1	5.39	4.82	89	5.23	97	4.56	85
2	8.80	8.21	93	8.27	94	7.67	87
3	10.58	11.61	110	11.10	105	10.48	99
**Mean %ER**			**97**		**99**		**90**
20°C							
1	5.39	4.51	84	4.47	83	2.56	47
2	8.80	7.86	89	6.70	76	6.01	68
3	10.58	9.76	92	8.57	81	7.87	74
**Mean %ER**			**88**		**80**		**63**

Going left to right, the columns contain information on measured concentrations of GFAP-C6 in serum samples placed at either 4°C or 20°C for: 0, 4, 24 and 48 hours. The %recovery from the 0 hour sample after increased time exposure to either 4°C og 20°C is depicted adjecent to each time slot. RE indicates recovery. T, time

**Table 6:** Inter- and Intra-assay variability of the GFAP-C6 ELISA.

Inter- and Intra-assay variability			
Sample name	GFAP-C6 (mean ng/mL)	Intra-assay variability%	Inter-assay variability%
Ctrl1	16.57	3.11	9.28
Ctrl2	7.31	4.97	8.91
QC1	8.21	3.56	10.23
QC2	10.25	2.70	7.20
QC3	5.88	3.61	11.80
QC4	4.10	8.49	15.92
QC5	16.29	2.86	7.71
**Mean**		**4.19**	**10.15**

Going left to right, the columns contain information on: mean concentrations of either selection peptide in buffer (Ctrl1 and Ctrl2) or GFAP-C6 in serum samples (QC1-5), Intra-assay variability and Inter-assay variability based on ten analytical runs.

**Table 7:** Technical specifications of the GFAP-C6 ELISA.

GFAP-C6 assay Technical parameters	
Detection range (LLOQ—ULOD)	2.16–247.38 ng/mL
Lower limit of detection (LLOD)	1.12 ng/mL
IC50	8.41 ng/mL
Intra-assay variation	5%
Inter-assay variation	11%
Dilution recovery	100±20%
Spiking recovery	100±20%
Recovery in Biotin low, high	102%, 102%
Recovery in Lipid low, high	93%, 90%
Recovery in Hemoglobin low, high	-, 93%
Analyte Stability, 4°C 4, 8, 48h	97%, 99%, 90%
Analyte Stability, 20°C 4, 8, 48h	88%, 80%, 63%
Freeze/Thaw, 1, 2, 3, 4 times	98%, 96%, 103%, 102%

Listed are the technical parameters of the GFAP-C6 ELISA. LLOQ indicates lower limit of quantification; ULOD, upper limit of detection; LLOD, lower limit of detection.

### Defining the TTM study and groups of good and unfavorable neurological outcome

At the Copenhagen University Hospital, 171 patients with CA were included in the TTM study. We had at least one serum sample available from 168 of these patients (98%). Levels of GFAP-C6 did not differ between those patients who had, or had not, experienced CPR from bystanders, had shockable or non-shockable initial rhythms or lactate levels above or below the median value of the total cohort at admission (S2 Fig). Further, GFAP-C6 levels did not correlate with time from CA to Return of Spontaneous Circulation (ROSC) at any of the blood sampling time points (S1 Table). Patients with a good neurological outcome were significantly younger (p = 0.0006), had a shorter time to ROSC (p = 0.001), lower lactate levels at time of admission (p = 0.0027) and more often presented with a shockable rhythm (p < 0.0001) than patients with an unfavorable neurological outcome (S2 Table). Generally similar to what others have previously observed [38–42], these results are as expected.

### GFAP-C6 levels in serum are elevated 72 hours after Cardiac arrest but do not correlate with neurological outcome

**Fig 3:**
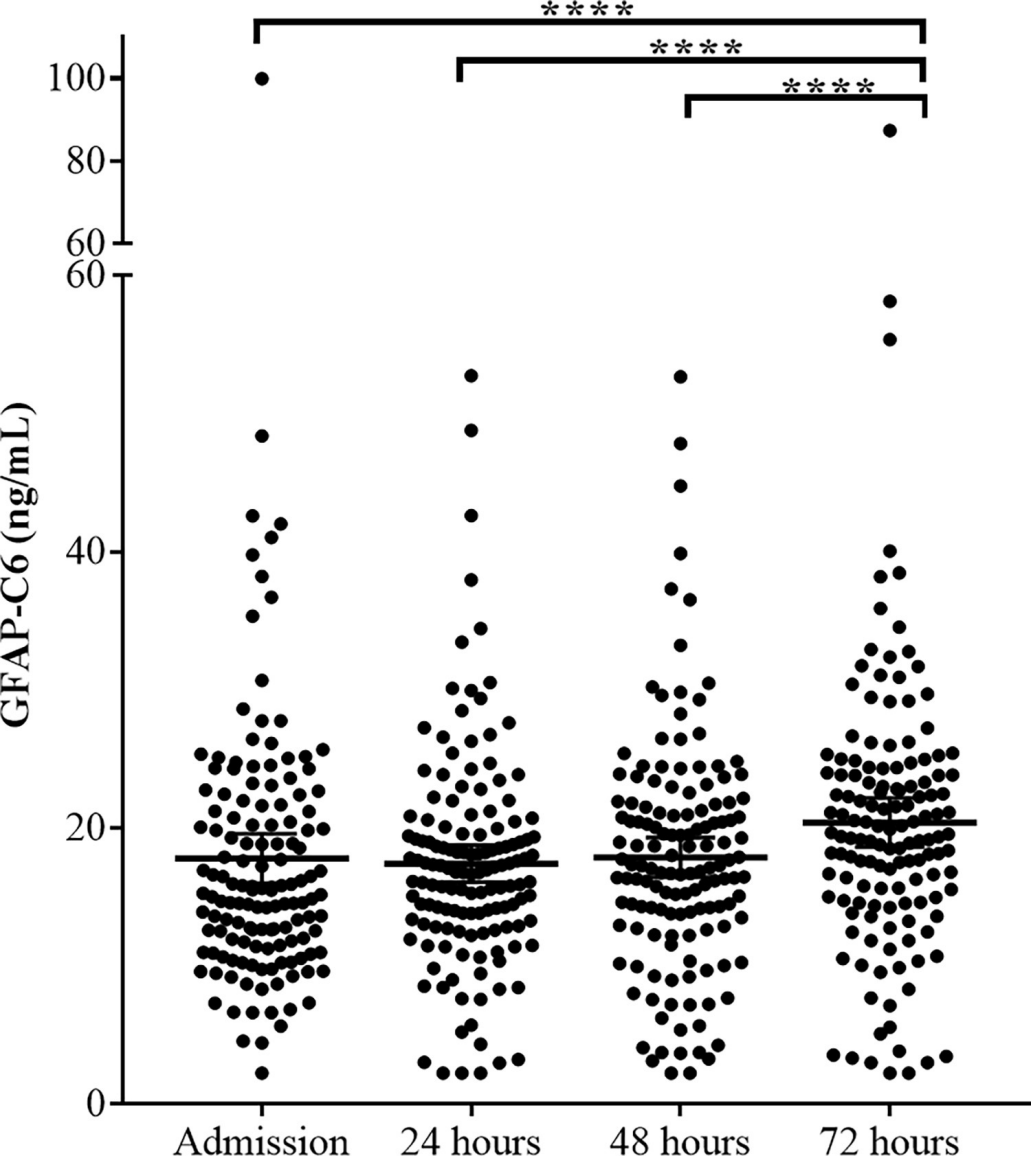
Serological levels of GFAP-C6 in CA patients at admission, 24, 48, and 72 hours after CA. Depicted in a scatter dot plot are levels of GFAP-C6 measured in the GFAP-C6 ELISA in serum of CA patients at admission, 24, 48, and 72 hours after CA. Error bars signify the mean with 95% confidence intervals. Significance of differences in biomarker levels is determined using One-way ANOVA with repeated measures. Adjustment for multiple comparisons was performed with Dunn's multiple comparisons test. **** indicates a P value below 0.0001.

**Fig 4:**
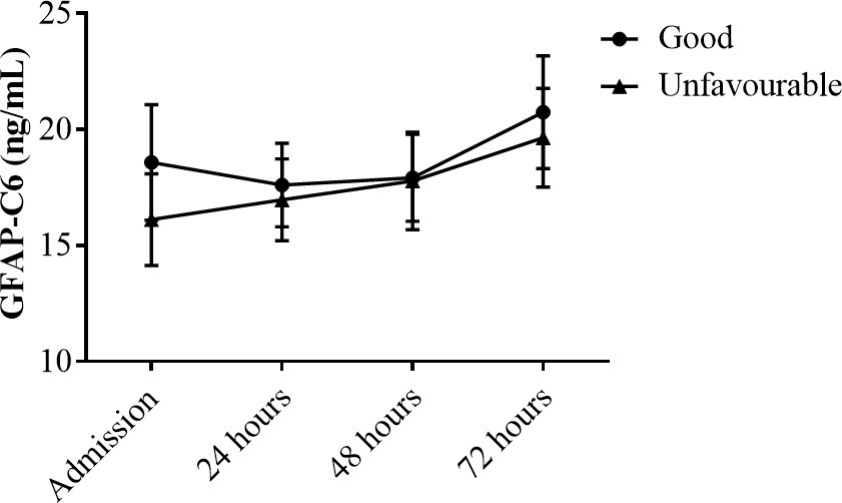
Serological levels of GFAP-C6 in CA patients of Good or Unfavorable neurological outcome at admission, 24, 48, and 72 hours after CA. Levels of GFAP-C6 measured in the GFAP-C6 ELISA in serum of CA patients of good or unfavorable neurological outcome at admission, 24, 48, and 72 hours after CA, are depicted as Line plots with error bars signifying the 95% confidence interval. Significance of differences in biomarker levels is determined using the Two-way ANOVA with repeated measures.

Levels of GFAP-C6 did not increase from time of admission to 24 hours or 48 hours after CA: Mean (SD) 17.79 (10.77) ng/mL, 17.40 (7.99) ng/mL (p > 0.99) and 17.87 (8.56) ng/mL (p = 0.35), respectively. On the other hand, a significant increase in GFAP-C6 levels was evident from time of admission as well as 24 hours and 48 hours after CA to 72 hours after CA: Mean (SD) 20.39 (10.59) (p < 0.0001). This increase suggests that caspase-6 cleaved GFAP may reflect processes related to CA and subsequent brain hypoxia (Fig 3). However, GFAP-C6 levels were not significantly different between patients of good versus unfavorable neurological outcome within the first 72 hours of CA: Mean (SD) 18.04 (12.06) ng/mL versus 15.87 (7.05) ng/mL, 17.59 (8.71) ng/mL versus 16.57 (5.99) ng/mL, 17.83 (9.30) ng/mL versus 17.44 (7.77) ng/mL and 20.90 (11.85) ng/mL versus 19.77 (7.61) ng/mL at admission, 24, 48 and 72 hours after CA, respectively (p_interaction_ = 0.36 and p_group_ = 0.48) (Fig 4).

### GFAP-C6 levels correlate with two other proteolytic fragments, tau-A and tau-C

**Fig 5:**
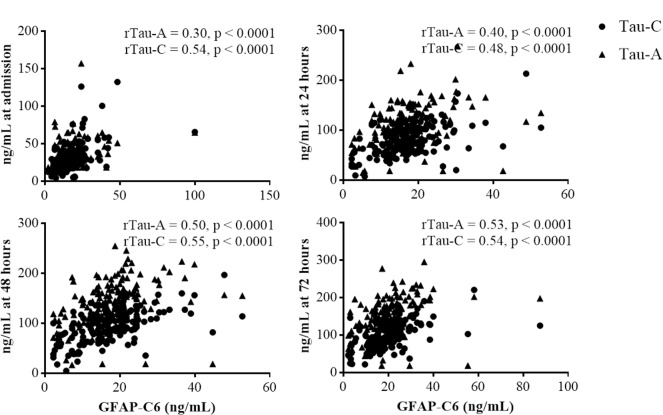
Correlations between GFAP-C6 levels and tau-A and tau-C levels at time of admission, 24, 48, and 72 hours after CA. Levels of GFAP-C6 measured in the GFAP-C6 ELISA in serum of CA patients are correlated to serological levels of the proteolytic fragments of tau, tau-A and tau-C at admission, 24, 48, and 72 hours after CA. Listed are the Spearman’s rho correlation coefficients, r, with the 95% confidence interval. P values represent the significance of correlation.

GFAP-C6 did not correlate to validated fluid biomarkers of brain injury such as neuron-specific enolase (NSE), S100B or total tau (T-tau) at any time point (S3 Table). Correlations to NSE were (r = 0.14; p = 0.09) 24 hours after CA, (r = 0.13; p = 0.13) 48 hours after CA and (r = 0.12; p = 0.21) 72 hours after CA. Correlations to S100B were (r = 0.07; p = 0.45) 24 hours after CA, (r = 0.05; p = 0.55) 48 hours after CA and (r = 0.02; p = 0.85) 72 hours after CA. Correlations to T-tau were (r = -0.04; p = 0.65) 24 hours after CA, (r = 0.05; p = 0.59) 48 hours after CA and (r = 0.00; p = 0.96) 72 hours after CA. Interestingly, modest correlations were observed between levels of GFAP-C6 and two proteolytic fragments of tau, tau-A and tau-C, but not total tau, at all time points (Fig 5 and S3 Table) possibly suggesting that proteolytic fragmentation reflects pathological processes not reflected by total protein levels. Correlations tau-A were (r = 0.30; p < 0.0001) at admission, (r = 0.40; p = < 0.0001) 24 hours after CA, (r = 0.50; p = < 0.0001) 48 hours after CA and (r = 0.53; p = < 0.0001) 72 hours after CA. Correlations to tau-C were (r = 0.54; p = < 0.0001) at admission, (r = 0.48; p = < 0.0001) 24 hours after CA, (r = 0.55; p = < 0.0001) 48 hours after CA and (r = 0.54; p = < 0.0001) 72 hours after CA. Further, GFAP-C6 levels at admission correlated with levels of C-reactive protein (r = 0.23; p = 0.0030) suggesting that the processes leading to caspase-6 mediated fragmentation of GFAP are to some extent connected with processes of the early immunologic response to CA (S3 Table).

## Discussion

In this study, we have presented a novel, competitive ELISA targeting caspase-6-cleaved GFAP in serum and evaluate the biological relevance of this neo-epitope in 168 CA patients with global cerebral ischemia. This is the first study specifically measuring the caspase-6 cleaved fragment of GFAP in serum. We showed that the GFAP-C6 ELISA provides reliable measurements and we observe increased levels of GFAP-C6 in serum from CA patients which suggests a possible relation of this biomarker to processes affected by CNS injury.

### The GFAP-C6 ELISA measures serum levels of GFAP-C6 with high precision and accuracy

To build the assay, we raised highly specific mAbs specifically recognizing the neo-epitope on GFAP fragments resulting from a cleavage at Asn^143^ by caspase-6. The ELISA was highly specific for its target, as shown using both synthetic peptides as well as caspase-6 cleaved rhGFAP. Performing spiking- and dilution recoveries, we found that a sample dilution of 1+1 provided both high precision and accuracy of measurements and allowed most samples to be within the measurement range of the assay. Additional investigations on precision and accuracy of the assay were performed using endogenous serum analytes, hemoglobin, lipid, and biotin, which may interfere with the binding of epitopes by antibodies or mimic the action of HRP causing falsely high or falsely low results [43–45]. We did not see any interference from these analytes suggesting that our measurements of GFAP-C6 are reliable across serum samples with different compositions of hemoglobin, lipids, and biotin. Furthermore, we assessed the implications of repeated freeze/thaw cycles of serum samples as well as exposure to different temperatures for various time intervals on biomarker levels as these factors are relevant in practice and may affect analyte stability [46–48]. We found no indication of alterations to the measured biomarker levels as a consequence of repeated freeze/thaw cycles or storage of serum at 4°C for up to 48 hours. On the other hand, serum samples placed at 20°C for 48 hours showed a mean decrease of GFAP-C6 levels of 37%. Taken together, these data suggest that for the GFAP-C6 ELISA, normal handling of serum samples does not pose a problem for reliable measurements of biomarker levels as long as care is taken not to expose the samples to room temperature for an excessive amount of time. Finally, the GFAP-C6 assay showed high technical reliability with inter- and intra assay variations well within limits of 15% and 10%, respectively. Altogether, we have produced a technically robust ELISA capable of quantifying the GFAP-C6 level in serum with high specificity, precision, and accuracy.

### Serum levels of GFAP-C6 increase 72 hours after CA, but do not predict neurological outcome

We see a significant increase in GFAP-C6 at 72 hours after CA suggesting that global cerebral ischemia affects processes related to the cleavage of GFAP by caspase-6. The literature on serological levels of GFAP is scarce. However, the few publications on the subject suggest that GFAP levels increase 24–48 hours after CA, showing differential levels between outcome groups at 12 hours after injury [10–12]. This is in line with a hypothetical model on the temporal profile of brain injury markers following CA, categorizing GFAP as a ‘sustained acute marker’ which increases in levels around 12 hours after CA, peaks at 24 hours and reaches pre-CA levels between 48–96 hours [49].

To our knowledge, this is the first investigation specifically targeting the caspase-6 cleaved GFAP fragment as a marker of brain injury. Based on previous observations on serological levels of full-length GFAP following CA, the increase of GFAP-C6 appears later. However, GFAP measurements in different CA cohorts can not be directly compared and future investigations into the temporal relation between full-length GFAP and GFAP-C6 would be of interest. There are several possible explanations as to why the caspase-cleaved GFAP fragment may show another temporal profile than its full-length counterpart. One possibility is that transport across the blood-brain-barrier differs between GFAP-C6 and full-length GFAP. This has been suggested to explain the lack of correlation between T-tau and two fragments of tau measured in serum obtained from the same CA cohort as applied in this study [50]. Another possibility is, that the cleavage of GFAP-C6 is a limiting factor in relation to how fast the fragment is generated. Only a few studies have investigated the effect of cerebral ischemia on the expression and activation of caspase-6 specifically. Following focal ischemic injury in rats, procaspase-6 shows moderate immunostaining in neurons and astrocytes within the stroke penumbra 12 hours after injury [32] and activation 24 hours after injury [51]. In a canine model of CA, however, activated caspase-6 appeared in brain homogenates as early as 10 minutes following reperfusion [52]. Thus, based on the few data available on the temporal profile of caspase-6 following ischemic brain injury, it does not appear that the cleavage of GFAP will limit the formation and subsequent release of GFAP-C6 compared to that observed for full-length GFAP.

Within the available timeframe of this study, GFAP-C6 serum levels are not indicative of neurological outcome at day 180 after CA. However, as the increase in GFAP-C6 serum levels is only initiating at 72 hours after injury, it is possible that GFAP-C6 levels are indeed reflecting pathological processes in the injured CNS and thus, levels at later time points may be indicative of neurological outcome. In the setting of CA, a possible prognostic value of GFAP-C6 levels later than 72 hours after CA is irrelevant as levels of the currently most used biochemical marker of prognosis, NSE, shows prognostic ability at 24 hours after CA [53,54]. However, in a setting of slowly evolving CNS damage, such as the pathology underlying dementias, GFAP-C6 serum levels might show diagnostic and/or prognostic abilities by reflecting processes affected by CNS injury, as suggested in this study. In support of this, several studies on CSF- and blood levels of total GFAP in patients of different dementia types, suggest that processes underlying neurodegenerative diseases affect the level of GFAP [55–60]. Also, caspase-6 activity has been associated with pathological processes underlying AD and is emerging as an early event that, if inhibited, may prevent progression of AD [61]. Thus, it is conceivable that caspase-6 cleavage of GFAP might add valuable information for the diagnosis and/or prognosis of dementia.

### GFAP-C6 correlates with two other brain protein fragments, tau-A and tau-C, but not total tau

GFAP-C6 did not correlate to validated fluid biomarkers of brain injury such as NSE, S100B or T-tau at any time point. However, levels of two proteolytic fragments of tau, tau-A and tau-C, previously shown to be associated with AD as well as severity of sports-related head trauma [62–65], showed a modest correlation with levels of GFAP-C6 at all time points. The lack of correlation between GFAP-C6 and markers of injury, T-tau and NSE, is surprising. However, levels of the two other proteolytic fragments, tau-A and tau-C, did not correlate with T-tau or NSE, either [50]. In light of these findings, future studies on the correlation between GFAP-C6 and its full-length counterpart, GFAP, could provide interesting knowledge on the biology behind GFAP-C6 formation and release. The lack of correlation between fragments GFAP-C6, tau-A, tau-C and full-length proteins, tau and NSE, might suggest that proteolytic fragmentation of proteins, as a consequence of CNS injury, reflects different processes than total level of protein. As tau-A and tau-C fragments have shown utility within diagnosis of AD and reflect severity of AD as well as severity of TBI, their modest correlation to GFAP-C6 provides some indication that GFAP-C6 might also provide information on pathological processes of the injured brain.

In conclusion, we developed a robust ELISA assay capable of quantifying serum levels of the caspase-6 cleaved fragment of GFAP, GFAP-C6, with high precision, and accuracy. We have shown that levels of this proteolytic fragment start to increase at 72 hours after CA and data of this study suggest a possible ability of GFAP-C6 to reflect pathological processes of the injured brain. Based on this preliminary investigation on the potential of GFAP-C6 as a serological marker of CNS injury, it would be of interest to investigate the biology behind formation and release of GFAP-C6 as well as its potential as a marker of other types of CNS injuries.

## Supporting information

S1 Fig Significant alignments of the neo-epitope sequence of GFAP-C6. Shown are the two sequences producing significant alignments to the neo-epitope sequence of GFAP-C6. The sequence was blasted for homology to other proteins using the “NPS@: Network Protein Sequence Analysis with the UniprotKB/Swiss-prot database” software online.
(TIF)

S2 Fig Association of CA characteristics with GFAP-C6 levels at admission, day 2, 3 and 4. Levels of GFAP-C6 measured in the GFAP-C6 ELISA in serum of CA patients at admission, day 2, 3 and 4, are depicted as dot plots with error bars signifying the 95% confidence interval. Compared at each time point is GFAP-C6 levels in those patients who had, or had not, experienced CPR from bystanders, had shockable or non-shockable initial rhythms and lactate levels above or below the median value of the total cohort at admission. Significance of differences in biomarker levels is determined using the Mann Whitney U test.
(TIF)

S1 Table Correlation between GFAP-C6 and Time from CA to ROSC. Listed are the Spearman’s rho correlation coefficients, r, with the 95% confidence interval. P values represents the significance of correlation.
(DOCX)

S2 Table Cardiac arrest data in groups of neurological outcome. Data are presented as mean±SD or median and lower to upper quartile (IQR) as appropriate. P value represents comparison between groups of good and unfavorable neurological outcome. CPR indicates cardiopulmonary resuscitation; ROSC, return of spontaneous circulation; min, minutes; mM, millimolar; n, number of patients.
(DOCX)

S3 Table Correlation between GFAP-C6 and other blood biomarkers. Listed are the Spearman’s rho correlation coefficients, r, with the 95% confidence interval. P values represents the significance of correlation. CA indicates cardiac arrest; tau-A, ADAM10 cleaved tau fragment; tau-C, caspase-3 cleaved tau fragment; HGB, hemoglobin; CRP, C-reactive protein; NSE, Neuron specific enolase; S100B, S100 calcium-binding protein B; T-tau, total tau; n, number of patients.
(DOCX)
