# Supplementary figures and images for: A caspase-6-cleaved fragment of Glial Fibrillary Acidic Protein as a potential serological biomarker of CNS injury after cardiac arrest
Source: PLoS One. 2019 Nov 6;14(11):e0224633. doi: 10.1371/journal.pone.0224633 (PMC6834260; doi:10.1371/journal.pone.0224633)

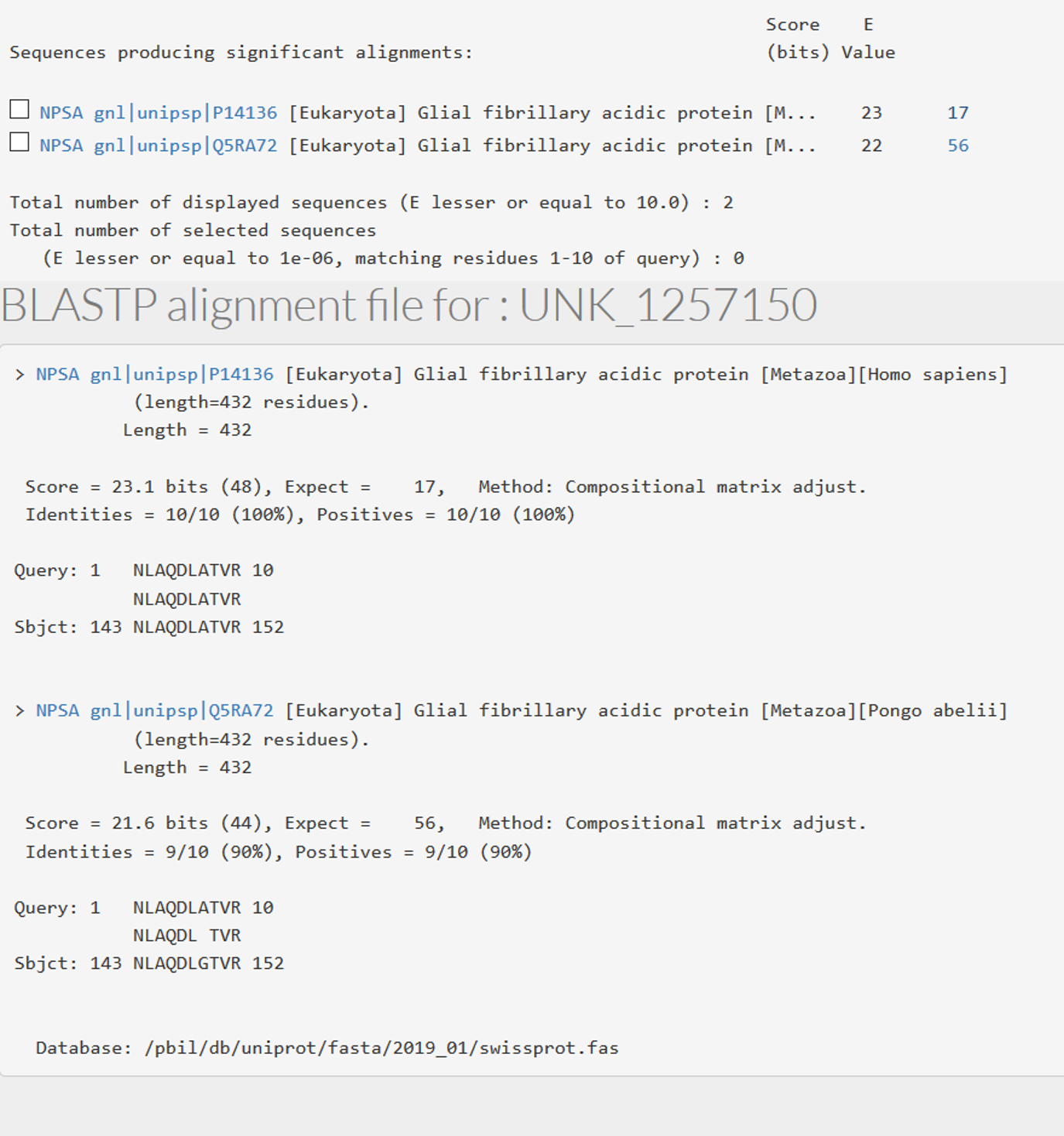

Supplement: S1 Fig — Shown are the two sequences producing significant alignments to the neo-epitope sequence of GFAP-C6. The sequence was blasted for homology to other proteins using the “NPS@: Network Protein Sequence Analysis with the UniprotKB/Swiss-prot database” software online. (TIF) [file pone.0224633.s001.tif]

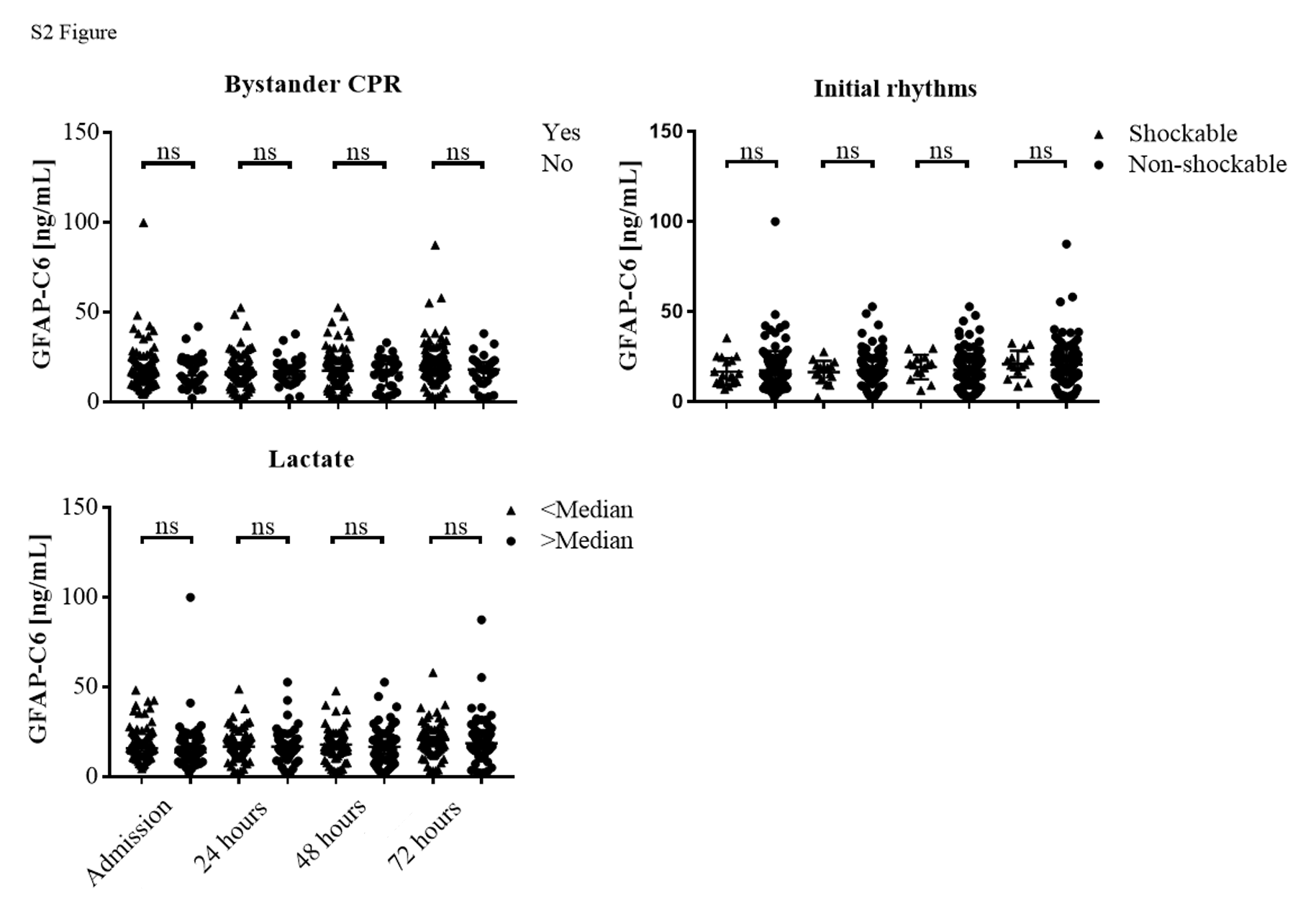

Supplement: S2 Fig — Levels of GFAP-C6 measured in the GFAP-C6 ELISA in serum of CA patients at admission, day 2, 3 and 4, are depicted as dot plots with error bars signifying the 95% confidence interval. Compared at each time point is GFAP-C6 levels in those patients who had, or had not, experienced CPR from bystanders, had shockable or non-shockable initial rhythms and lactate levels above or below the median value of the total cohort at admission. Significance of differences in biomarker levels is determined using the Mann Whitney U test. (TIF) [file pone.0224633.s002.tif]
