# Supplementary material for: A caspase-6-cleaved fragment of Glial Fibrillary Acidic Protein as a potential serological biomarker of CNS injury after cardiac arrest
Source: PLoS One. 2019 Nov 6;14(11):e0224633. doi: 10.1371/journal.pone.0224633 (PMC6834260; doi:10.1371/journal.pone.0224633)
Supplement: S1 Table — Listed are the Spearman’s rho correlation coefficients, r, with the 95% confidence interval. P values represents the significance of correlation. (DOCX) [file pone.0224633.s003.docx]

S1 table. **Correlation between GFAP-C6 and Time from CA to ROSC.**

| **Time from CA to ROSC** | **GFAP-C6** | |
| --- | --- | --- |
|  | R (95% CI) | p-value |
| Admission (n = 163) | -0.07 (-0.23-0.09) | 0.363 |
| 24 hours (n = 154) | -0.10 (-0.26-0.06) | 0.218 |
| 48 hours (n = 153) | -0.07 (-0.23-0.10) | 0.411 |
| 72 hours (n = 148) | -0.02 (-0.18-0.15) | 0.853 |
|  |  |  |

Listed are the Spearman’s rho correlation coefficients, r, with the 95% confidence interval. P values represents the significance of correlation.
