# Supplementary material for: A caspase-6-cleaved fragment of Glial Fibrillary Acidic Protein as a potential serological biomarker of CNS injury after cardiac arrest
Source: PLoS One. 2019 Nov 6;14(11):e0224633. doi: 10.1371/journal.pone.0224633 (PMC6834260; doi:10.1371/journal.pone.0224633)
Supplement: S2 Table — Data are presented as mean±SD or median and lower to upper quartile (IQR) as appropriate. P value represents comparison between groups of good and unfavorable neurological outcome. CPR indicates cardiopulmonary resuscitation; ROSC, return of spontaneous circulation; min, minutes; mM, millimolar; n, number of patients. (DOCX) [file pone.0224633.s004.docx]

S2 table. **Cardiac arrest data in groups of neurological outcome.**

|  | Good outcome | Unfavourable outcome | p-value |
| --- | --- | --- | --- |
|  | (n=104) | (n=67) |  |
| **Cardiac arrest characteristics:** |  |  |  |
| - Bystander CPR - n (%) | 85 (82) | 52 (78) | 0.560 |
| - Shockable initial rhythm - n (%) | 100 (97) | 52 (78) | <0.0001 |
| - Time to ROSC - min. (IQR) | 19 (12–27) | 26 (18–39) | 0.0001 |
| - Lactate at admission - mM. (IQR) | 6 (3-9) | 9 (5-13) | 0.003 |
|  |  |  |  |

Data are presented as mean±SD or median and lower to upper quartile (IQR) as appropriate. P value represents comparison between groups of good and unfavorable neurological outcome. CPR indicates cardiopulmonary resuscitation; ROSC, return of spontaneous circulation; min, minutes; mM, millimolar; n, number of patients.
