# Supplementary material for: A caspase-6-cleaved fragment of Glial Fibrillary Acidic Protein as a potential serological biomarker of CNS injury after cardiac arrest
Source: PLoS One. 2019 Nov 6;14(11):e0224633. doi: 10.1371/journal.pone.0224633 (PMC6834260; doi:10.1371/journal.pone.0224633)
Supplement: S3 Table — Listed are the Spearman’s rho correlation coefficients, r, with the 95% confidence interval. P values represents the significance of correlation. CA indicates cardiac arrest; tau-A, ADAM10 cleaved tau fragment; tau-C, caspase-3 cleaved tau fragment; HGB, hemoglobin; CRP, C-reactive protein; NSE, Neuron specific enolase; S100B, S100 calcium-binding protein B; T-tau, total tau; n, number of patients. (DOCX) [file pone.0224633.s005.docx]

S3 table. **Correlation between GFAP-C6 and other blood biomarkers.**

|  | **GFAP-C6** | |
| --- | --- | --- |
|  | R (95% CI) | p-value |
| **Admission** |  |  |
| Tau-A (n = 163) | 0.30 (0.15-0.44) | < 0.0001 |
| Tau-C (n = 163) | 0.54 (0.42-0.64) | < 0.0001 |
| HGB (n = 163) | 0.07 (-0.09-0.23) | 0.366 |
| CRP (n = 162) | 0.23 (0.08-0.38) | 0.003 |
| **24 hours after CA** |  |  |
| Tau-A (n = 154) | 0.40 (0.25-0.53) | < 0.0001 |
| Tau-C (n = 154) | 0.48 (0.35-0.60) | < 0.0001 |
| HGB (n = 152) | 0.05 (-0.12-0.21) | 0.540 |
| CRP (n = 152) | 0.05 (0.12-0.21) | 0.579 |
| NSE (n = 142) | 0.14 (-0.3-0.31) | 0.091 |
| S100B (n = 142) | 0.07 (-0.11-0.24) | 0.447 |
| T-tau (n = 152) | -0.04 (-0.21-0.13) | 0.624 |
| **48 hours after CA** |  |  |
| Tau-A (n = 152) | 0.50 (0.37-0.62) | < 0.0001 |
| Tau-C (n = 152) | 0.55 (0.42-0.65) | < 0.0001 |
| HGB (n = 148) | 0.07 (-0.10-0.24) | 0.393 |
| CRP (n = 148) | 0.06 (0.11-0.22) | 0.509 |
| NSE (n = 132) | 0.13 (-0.05-0.30) | 0.551 |
| S100B (n = 132) | 0.05 (-0.13-0.23) | 0.551 |
| T-tau (n = 149) | 0.05 (-0.12-0.21) | 0.586 |
| **72 hours after CA** |  |  |
| Tau-A (n = 148) | 0.53 (0.39-0.64) | < 0.0001 |
| Tau-C (n = 148) | 0.54 (0.41-0.65) | < 0.0001 |
| HGB (n = 139) | 0.07 (-0.10-0.24) | 0.393 |
| CRP (n = 139) | -0.01 (-0.19-0.16) | 0.864 |
| NSE (n = 124) | 0.12 (-0.07-0.29) | 0.210 |
| S100B (n = 124) | 0.02 (-0.17-0.20) | 0.846 |
| T-tau (n = 145) | 0.004 (-0.17-0.17) | 0.961 |

Listed are the Spearman’s rho correlation coefficients, r, with the 95% confidence interval. P values represents the significance of correlation. CA indicates cardiac arrest; tau-A, ADAM10 cleaved tau fragment; tau-C, caspase-3 cleaved tau fragment; HGB, hemoglobin; CRP, C-reactive protein; NSE, Neuron specific enolase; S100B, S100 calcium-binding protein B; T-tau, total tau; n, number of patients.
